# Nanostructured germanium deposited on heated substrates with enhanced photoelectric properties

**DOI:** 10.3762/bjnano.7.142

**Published:** 2016-10-21

**Authors:** Ionel Stavarache, Valentin Adrian Maraloiu, Petronela Prepelita, Gheorghe Iordache

**Affiliations:** 1 National Institute of Materials Physics, 405A Atomistilor Street, 077125 Magurele, Ilfov, Romania; 2 National Institute for Laser, Plasma and Radiation Physics, 409 Atomistilor Street, 077125 Magurele, Ilfov, Romania

**Keywords:** germanium nanoparticle, photocurrent, photodetectors, response time, transport mechanism

## Abstract

Obtaining high-quality materials, based on nanocrystals, at low temperatures is one of the current challenges for opening new paths in improving and developing functional devices in nanoscale electronics and optoelectronics. Here we report a detailed investigation of the optimization of parameters for the in situ synthesis of thin films with high Ge content (50 %) into SiO_2_. Crystalline Ge nanoparticles were directly formed during co-deposition of SiO_2_ and Ge on substrates at 300, 400 and 500 °C. Using this approach, effects related to Ge–Ge spacing are emphasized through a significant improvement of the spatial distribution of the Ge nanoparticles and by avoiding multi-step fabrication processes or Ge loss. The influence of the preparation conditions on structural, electrical and optical properties of the fabricated nanostructures was studied by X-ray diffraction, transmission electron microscopy, electrical measurements in dark or under illumination and response time investigations. Finally, we demonstrate the feasibility of the procedure by the means of an Al/n-Si/Ge:SiO_2_/ITO photodetector test structure. The structures, investigated at room temperature, show superior performance, high photoresponse gain, high responsivity (about 7 AW^−1^), fast response time (0.5 µs at 4 kHz) and great optoelectronic conversion efficiency of 900% in a wide operation bandwidth, from 450 to 1300 nm. The obtained photoresponse gain and the spectral width are attributed mainly to the high Ge content packed into a SiO_2_ matrix showing the direct connection between synthesis and optical properties of the tested nanostructures. Our deposition approach put in evidence the great potential of Ge nanoparticles embedded in a SiO_2_ matrix for hybrid integration, as they may be employed in structures and devices individually or with other materials, hence the possibility of fabricating various heterojunctions on Si, glass or flexible substrates for future development of Si-based integrated optoelectronics.

## Introduction

In the recent years, much attention was directed to study quantum confinement in nanostructures. Through the influence of quantum confinement on the electrical and optical properties new paths to improving and develop functional devices in nanoscale electronics and optoelectronics can be opened. This is closely related to the increase of high-speed operation, good reliability, low power consumption and the decrease of unit price that led to the rapid development of the semiconductor device market and to the continuous downscaling of devices. Regarding the downscaling process, high mobility, good process compatibility with the well-developed CMOS technology, and the extension of the photosensitivity range to the near-infrared (NIR) are the properties that make devices based on Ge nanoparticles (Ge-nps) promising candidates to substitute or to improve the conventional Si-based devices [1–7]. However, due to the lower binding energy of Ge atoms in comparison to Si atoms, Ge-nps can be formed in samples annealed at significantly lower synthesis temperatures of 600–900 °C compared to around 1100 °C for Si [8–9].

Regarding an application at the nanoscale, problems related to the indirect bandgap issue of Ge-based materials are partially overcome, but the difficulty of controlling size and shape of the nanoparticles still remains. New approaches for fine-tuning the size and shape of nanoparticles would facilitate the understanding of their quantum confinement behavior and a more accurate evaluation of their performance. For example, the electrical behavior and spectral response of nanostructures based on Ge-nps depend not only on the Ge-nps size but also on the embedding matrix [10], the density of the nanoparticles [11], the presence of surface or interface states [12], the amorphous or crystalline state of the nanostructure [13], the shape of the nanostructure and whether or not it is layered [14]. Taking into account the influence of all these factors, the conduction or light absorption mechanism in the nanostructures appear to be quite complex, and it cannot be fully described only through Ge-nps size.

Ge-nps embedded in different dielectric matrices (e.g., SiO_2_, Si_3_N_4_ or HfO_2_) have been already used for fabrication of high-efficiency photodetectors [15], multilayer memory devices [16] and other applications as solar photoconversion cells [17], batteries [18] and biosensors [19].

To produce high-quality Ge particles packed into different matrices, various approaches are reported in scientific papers such as pulsed laser deposition [20–21], sol–gel [22], evaporation under vacuum [23], chemical vapor deposition [24], microwave-assisted heating [25], implantation [26], RF magnetron sputtering [27]. However, for most of these approaches, thermal treatments were necessary after the deposition process in order to obtain high-quality nanostructures based on crystalline Ge [28].

The most important parameter to be finely tuned is the substrate temperature during deposition. The formation of Ge-nps is much more difficult than that of Si nanoparticles due to the larger diffusivity of Ge at high temperatures. GeO_2_ is thermodynamically less stable than SiO_2_ and the formation of GeO (gas) leads to the decrease of Ge concentration in the host matrix, a higher concentration of defect states and a mechanical stress induced in the system by the difference between the thermal expansion coefficients of Ge and SiO_2_ and the large lattice constant of Ge [29–32]. The high temperature used for the synthesis excludes the use of low-cost materials such as flexible or glass substrates resulting in a higher cost of fabrication.

In this paper, we report a detailed investigation of the optimization of parameters for the in situ synthesis of thin films with high Ge content into SiO_2_. The Ge-nps were directly formed during co-deposition of SiO_2_ and Ge on substrates at 300, 400 and 500 °C, and no further thermal treatments were necessary after the deposition process. The paper also reports the influence of film structure on electrical and photoelectrical properties. Finally, we demonstrate the feasibility of the procedure by means of an Al/n-Si/Ge:SiO_2_/ITO photodetector test structure. The obtained photoresponse gain is attributed mainly to the Ge-nps packed into the SiO_2_ matrix and to the conditions during sample preparation, showing the direct connection between synthesis and opto-electrical properties of the nanostructures. The structures, investigated at room temperature, show superior performance, such as high responsivity, fast response time and great optoelectronic conversion efficiency over a wide operation bandwidth. Our deposition approach emphasizes the great potential of Ge-nps embedded in SiO_2_ thin films for hybrid integration, as they may be employed in structures and devices individually or with other materials. This yields the possibility of fabricating various heterojunctions on Si for the future development of Si-based integrated optoelectronics.

## Results and Discussion

In this section the results on structural, electrical and photoelectrical properties of Ge-nps embedded in SiO_2_ thin film are summarized. The influence of the temperature on the photodetector test structure, fabricated on substrates at 300, 400 and 500 °C is also described.

**Figure 1:**
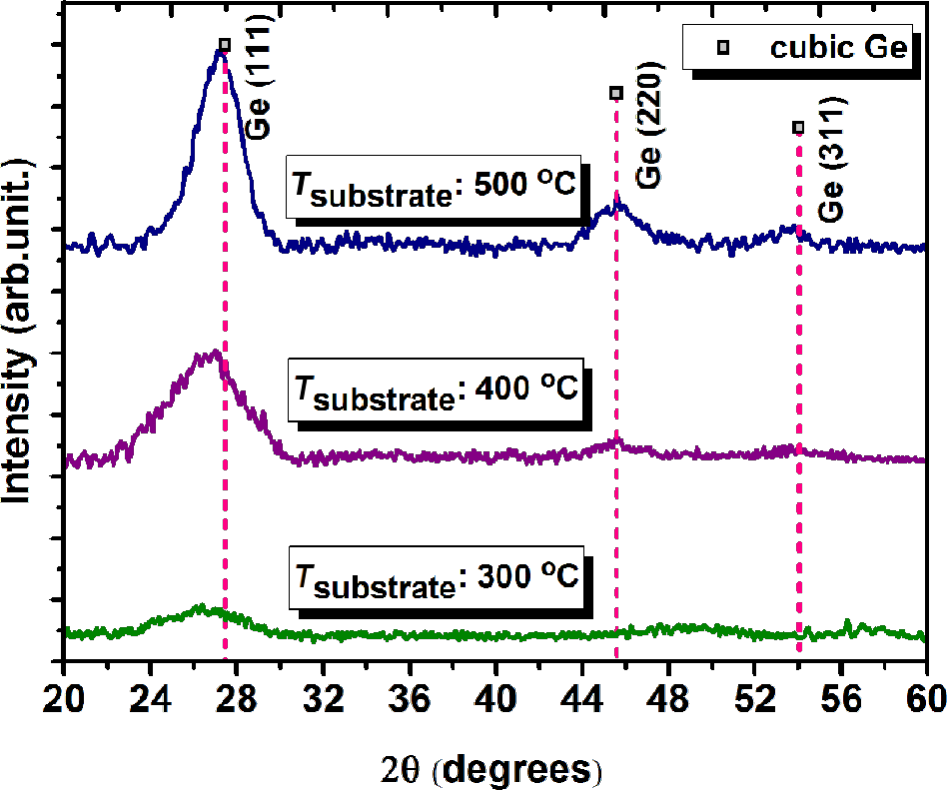
X-ray diffractograms of Ge:SiO_2_ thin films deposited at 300, 400 and 500 °C.

In Figure 1, the diffractograms recorded of thin films deposited by RF magnetron sputtering on substrates at 300, 400 and 500 °C are presented. There are also represented the X-ray diffraction (XRD) patterns of cubic Ge (ICSD no. 79-0001). Analyzing the obtained diffractograms, it is obvious that Ge:SiO_2_ films deposited at 300 °C are amorphous, while the films deposited at higher temperatures (400 and 500 °C) have a crystalline structure. The diffractograms of the films deposited at 400 and 500 °C show a clear main reflection corresponding to cubic Ge(111) and two smaller maxima corresponding to cubic Ge(220) and cubic Ge(311). The average Ge-nps size of about 5 nm formed in thin films deposited at 500 °C was estimated using the Debye–Scherrer equation. An average difference of about 1 nm in Ge-nps diameter was observed for films deposited at 500 °C and films deposited at 400 °C. The main peaks are slightly shifted to smaller angles compared to the ICSD patterns and these shifts decrease as the annealing temperature increases. The shift of peaks position is widely debated in scientific literature and it is partly attributed to the decomposition of the mixed oxide of Ge and Si, (Ge,Si)O_2_, which helps to reduce tensile strain in the layer [30,33].

**Figure 2:**
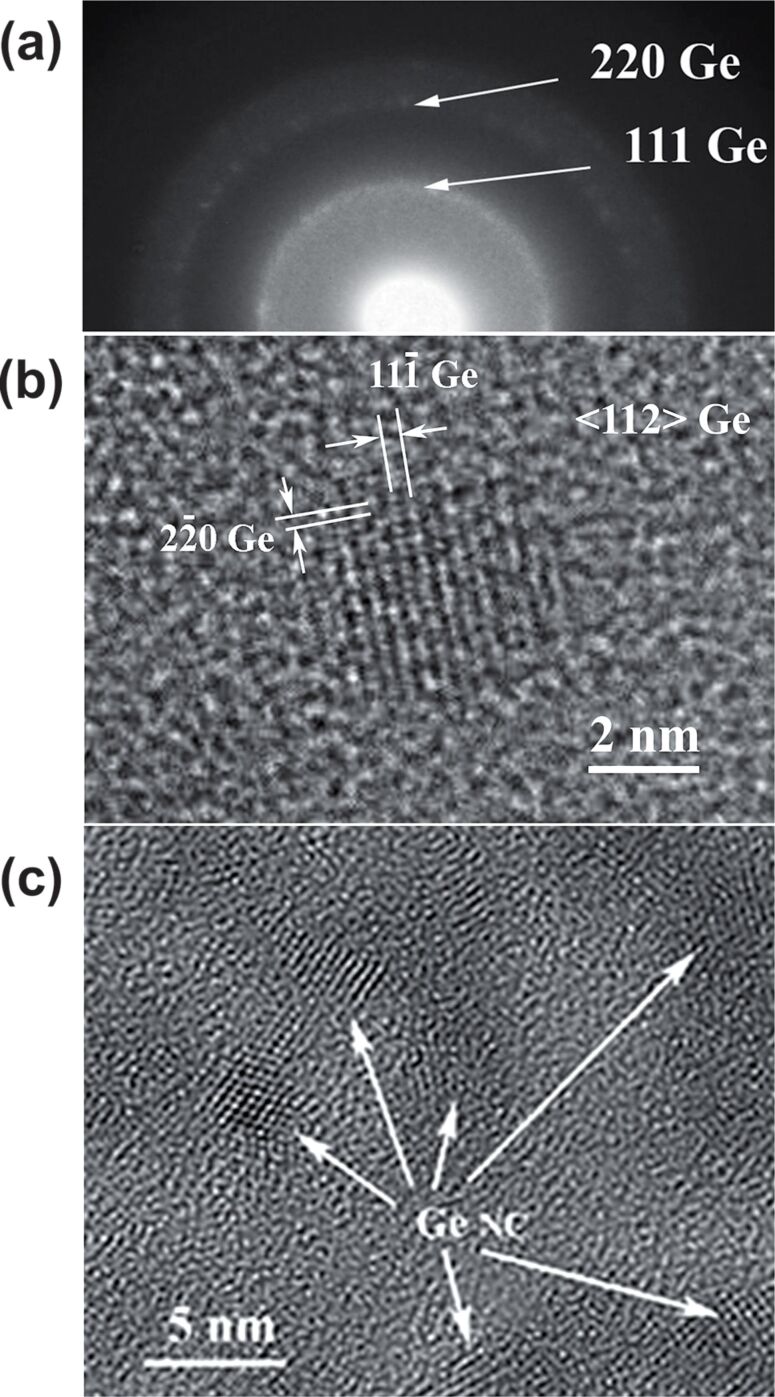
HRTEM images of the Ge:SiO_2_ thin film co-deposited on a Si substrate at 500 °C: (a) corresponding SAED pattern; (b) close-up of a Ge-np within the SiO_2_ thin film; (c) spatial distribution of Ge-nps in the oxide matrix.

In Figure 2, the results of high resolution transmission electron microscopy (HRTEM) investigations of the Ge:SiO_2_ thin films deposited on Si substrates at 500 °C are presented. Selected area electron diffraction (SAED) (Figure 2a) and HRTEM (Figure 2b) demonstrate the <112> orientation of Ge-nps. The HRTEM image in Figure 2c shows the spatial distribution of the Ge-nps in the oxide matrix. The main reflections measured on the central part of the SAED pattern correspond to cubic Ge(111) and cubic Ge(220) (ICSD no. 79-0001) confirming the existence of the crystalline phase. HRTEM images are used to estimate the average size of Ge-nps (around 5 nm in this case). Figure 2c reveals that Ge-nps are randomly distributed in the SiO_2_ matrix. The results of electron microscopy investigations performed on films deposited at 500 °C are in good agreement with the XRD measurements.

**Figure 3:**
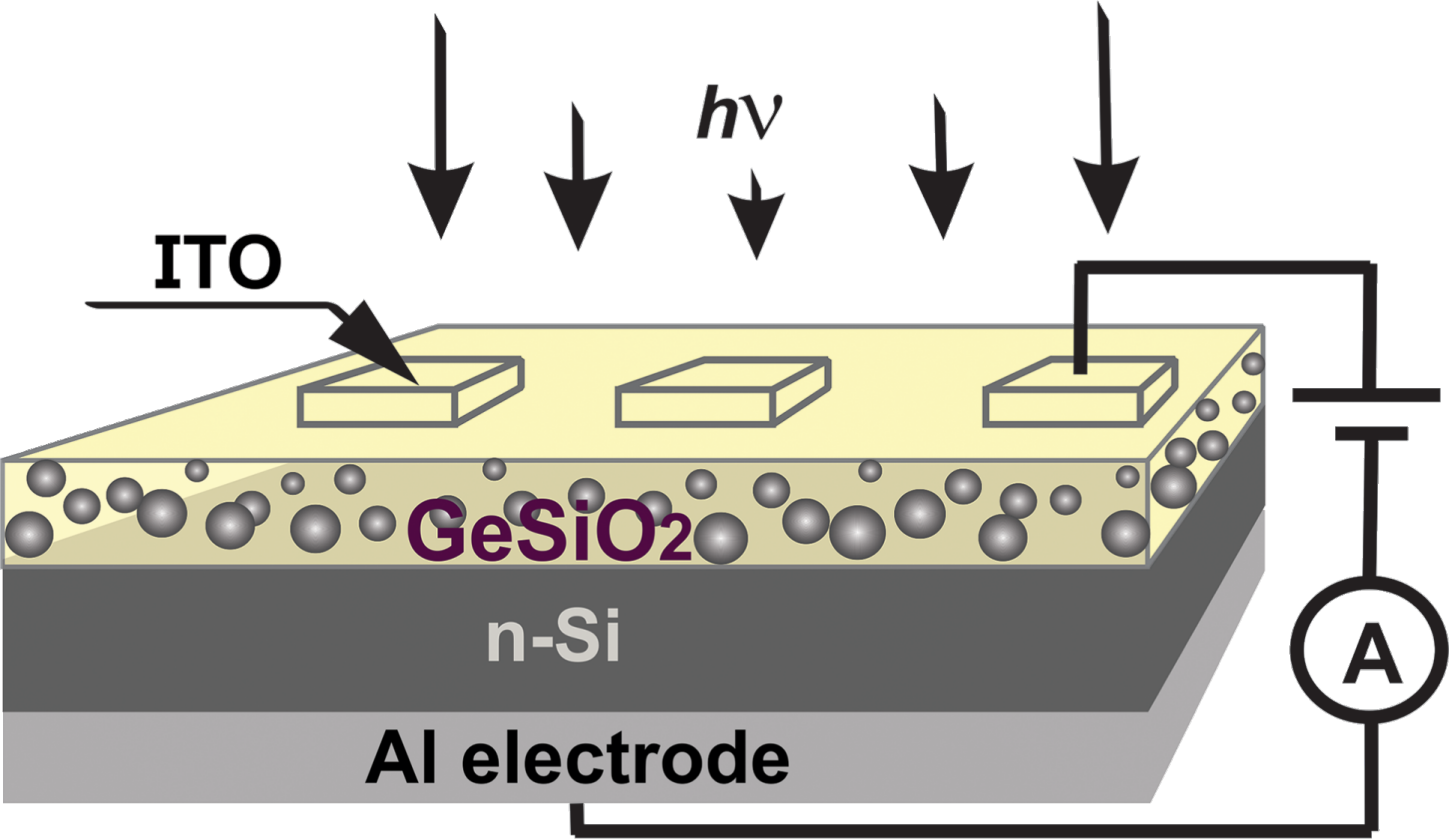
Schematic of sample structure and electrical measurement.

To test the photosensitivity properties of the material employed in the photodetector test structure, current density versus voltage (*J*–*V*) measurements, in dark and under integral light illumination were recorded in CMOS configuration. In the drawing presented in Figure 3, the measurement set-up and sample structure are described schematically. The active area of the test photodetector is 9 mm^2^.

**Figure 4:**
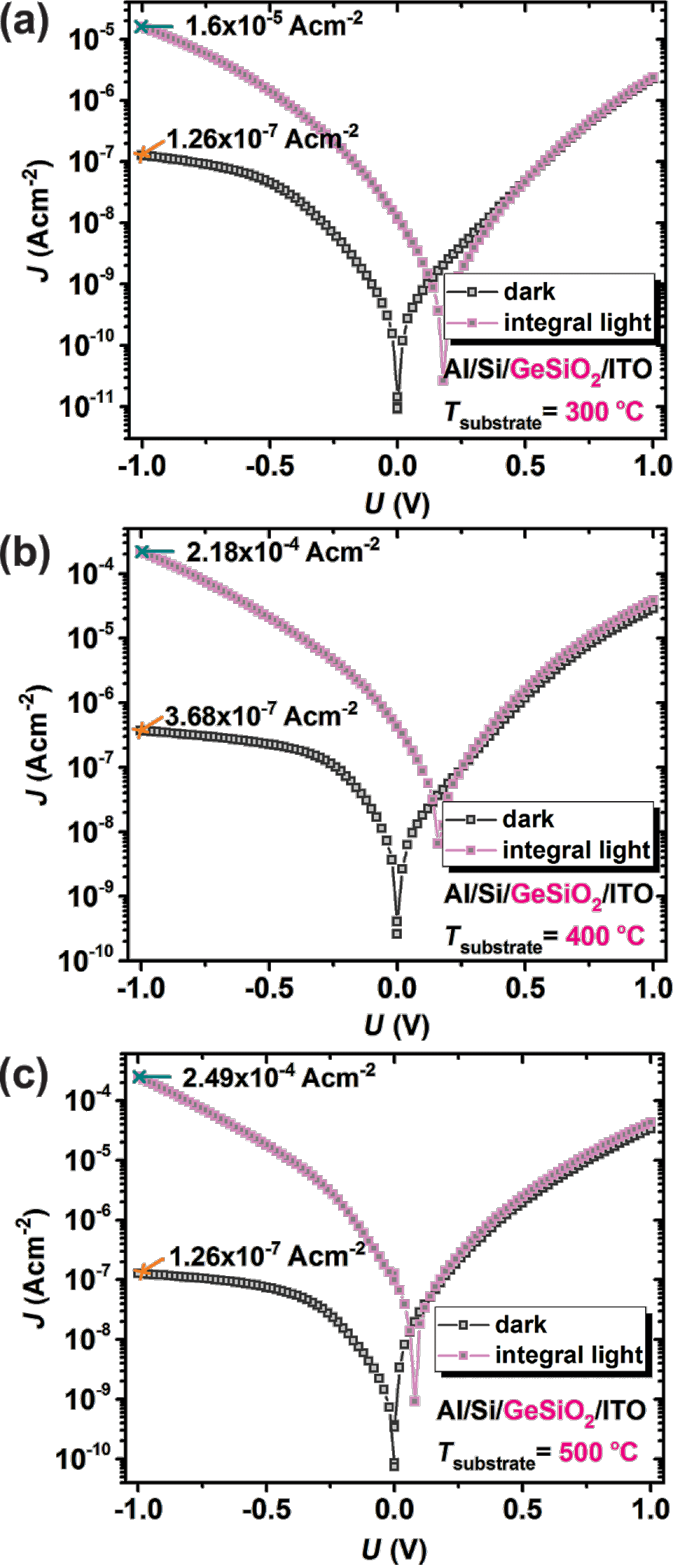
Current density versus voltage characteristics in dark (empty squares) or under integral light (filled squares). Characteristics recorded from structures with Ge-nps incorporated into SiO_2_ thin films deposited at different temperatures (a) 300 °C; (b) 400 °C; (c) 500 °C.

Characteristics recorded on structures containing Ge-nps in the SiO_2_ matrix, deposited at temperatures of 300, 400 and 500 °C, are presented in Figure 4. As one can see, they show a significant increase of the photoresponse as the temperature during the deposition increases. However, a smaller gap difference is observed between the curves corresponding to structures obtained at 400 and 500 °C. This suggests that the optimum substrate temperature is around 500 °C. For all the investigated test structures voltages between −1 V to 1 V with 20 mV steps were applied on the ITO contact while the n-Si substrate was grounded. In this configuration, the bias voltage is applied across the series combination of junctions, namely at the ITO/Ge:SiO_2_ interface, the Ge:SiO_2_ film region, and the junction situated at Ge:SiO_2_/Si interface [34].

The rectifying behavior (10^2^ rectification ratio at 1 V) observed in the absence of light can be the result of the serial combination of these interface junctions that can act as rectifying diode-like contacts. It is known that the chemical reduction of GeO*_x_* plays an important role and represents the major mechanism to produce size-controlled Ge-nps, embedded into a dielectric matrix of stoichiometric SiO_2_. This determines the dark current level (which should be as small as possible) whereas Ge:SiO*_x_* layers with small oxygen deficit (*x* < 2) represent the favorable components for photovoltaic applications, according to the analysis conducted by A. Nyrow and co-workers [35]. This approach is reported also in the research led by W. Little et al. which assigns the light emission in Ge-nps to the presence of oxygen-terminated nanoparticles [36].

**Figure 5:**
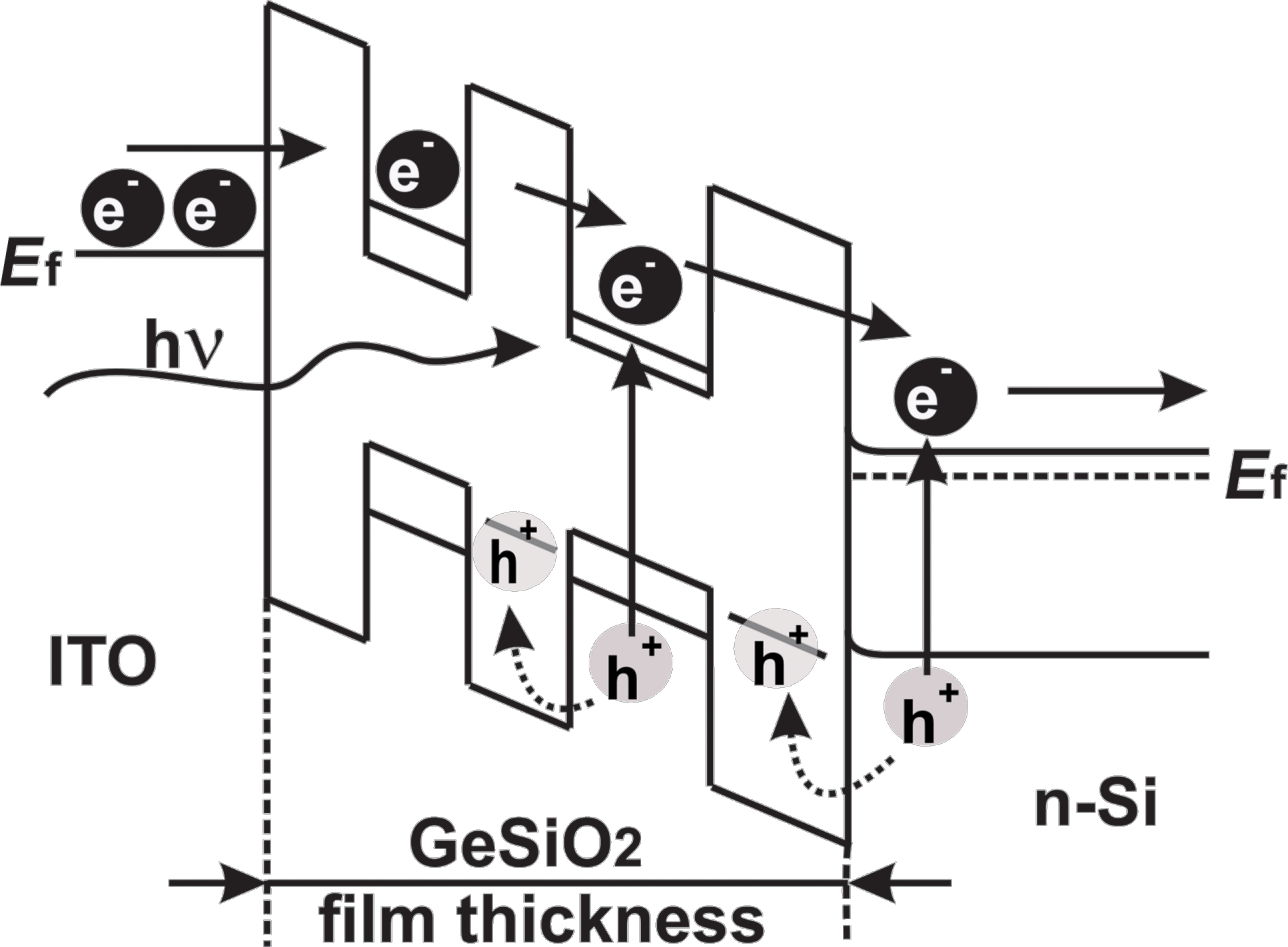
The charge carriers transport mechanism described schematically.

In Figure 5, the transport mechanism that takes place in this kind of structures is described schematically. In darkness, the current behavior of the Al/n-Si/Ge:SiO_2_/ITO structure (schematically presented in Figure 5) is determined by the electrons tunneling from the ITO top electrode into the Ge-nps located in the SiO_2_ oxide layer and the electron transport in Ge:SiO_2_ film by a tunneling mechanism between neighboring Ge-nps [9,37]. The investigated (Ge-nps):SiO_2_ system behaves like a resistor network where each Ge-np is connected with its neighboring Ge-nps by a finite tunneling resistor. In this way, the activated carriers in the Ge-nps would tunnel to the nearest Ge-nps, following the path with the lowest resistance. A rather similar transport mechanism was suggested as a result of the analysis conducted by B. C. Hsu and co-workers [38]. When the photodetector test structure is illuminated with integral light at reverse bias, a significant increase of the current density by a factor of about 10^3^ is observed as a result of the separation of electron–hole pairs generated in the Ge-nps and the Si substrate. The obtained photoresponse gain factor increases from about 10^2^ (at 300 °C) to about 10^3^ (at 500 °C) with the temperature increase during deposition.

Under illumination, electrons and holes are generated in the Ge-nps and in the Si substrate and they move by tunneling between neighboring Ge-nps. During transport, the positively charged holes are dynamically trapped within the Ge-nps incorporated into SiO_2_ matrix improving the electron injection, leading to an increase of negative photoconductivity [39]. Under forward bias, the characteristics remain roughly unaffected.

**Figure 6:**
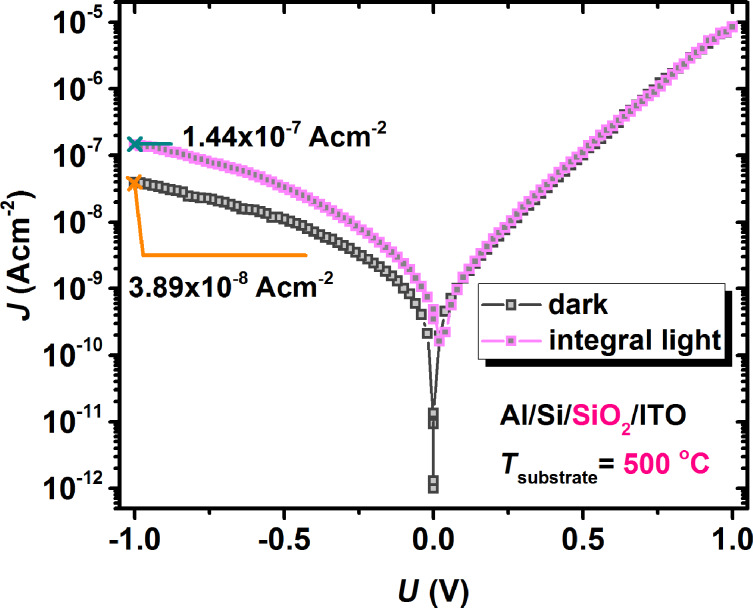
Current density versus voltage characteristics in dark (empty squares) or under integral light (filled squares) conditions of a structure without Ge-nps.

Finally, to test the influence of Ge-nps, a reference structure was deposited under the same conditions as above, maintaining the total thickness of the oxide film and the structure, but without Ge-nps incorporated into SiO_2_. The characteristic with the largest increase of the photoresponse was observed for the reference structure fabricated at 500 °C and it is presented in Figure 6. It shows a very small increase of the current density under illumination compared with dark conditions. The much higher photosensitivity of the test photodetector-like structures with Ge-nps embedded in SiO_2_, under normal environment conditions, reveals the essential role of the Ge-nps in dramatically improving the electro-optical parameters of the structure.

**Figure 7:**
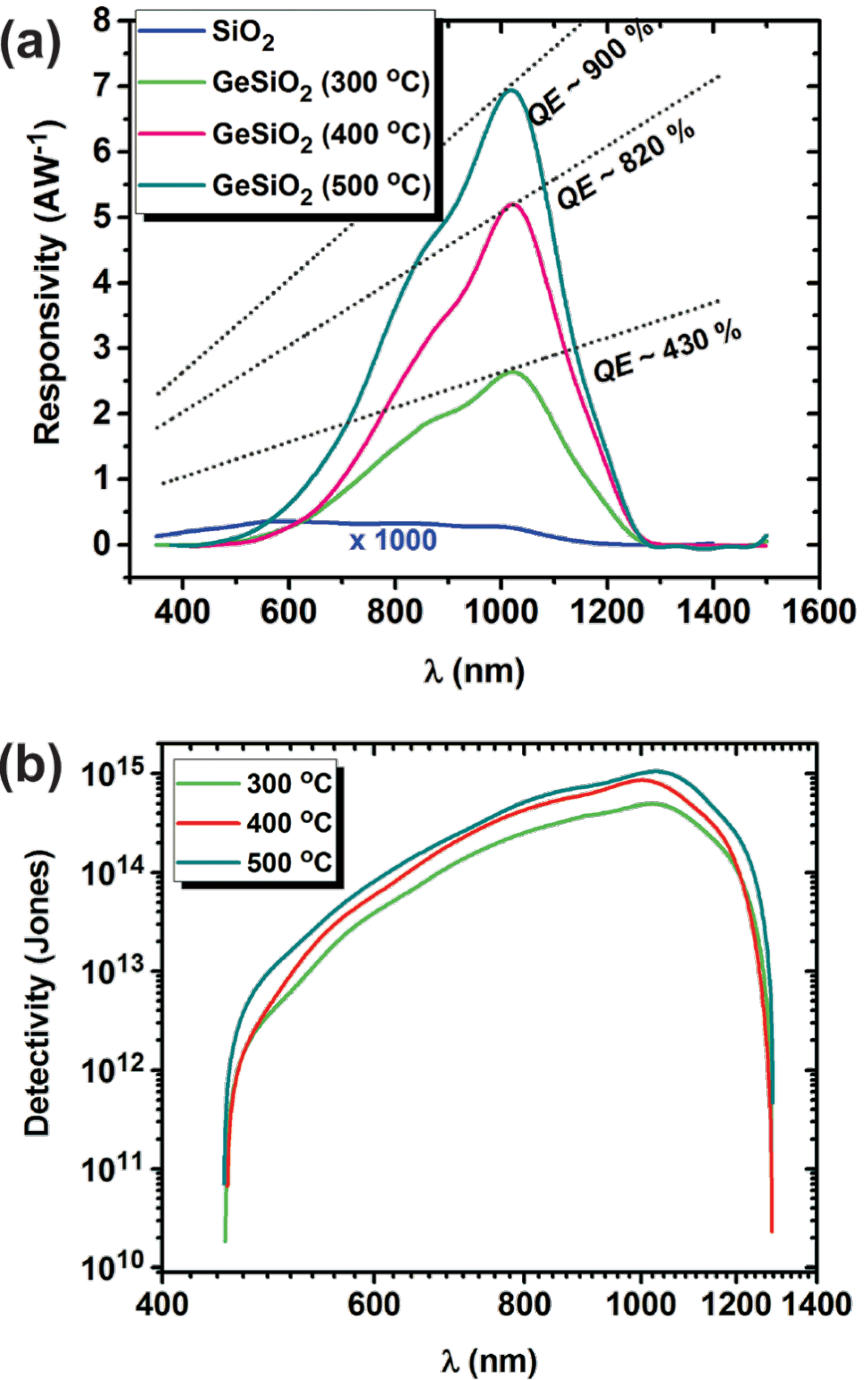
Photodetector responsiveness: a) spectral photoresponsivity, for Al/n-Si/Ge:SiO_2_/ITO and Al/n-Si/SiO_2_/ITO structures deposited on substrates at different temperatures, under monochromatic light and −1 V external bias respectively; b) spectral detectivity for Al/n-Si/Ge:SiO_2_/ITO structures obtained at different temperatures.

Figure 7a presents the spectral photoresponsivity *R*_spectral_ calculated for −1 V bias over a wide range of incident light wavelengths (350–1500 nm) for Al/n-Si/Ge:SiO_2_/ITO structures with the Ge:SiO_2_ layer deposited at 300, 400 and 500 °C. The calculated value of *R*_spectral_ represents the ratio between the photogenerated current and the incident optical power (*P*_in_) and it was obtained using the following equation [40]:

[1]
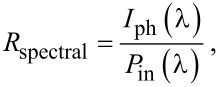


where *I*_ph_ is the measured photocurrent under illumination and *P*_in_ is the optical power incident on the active area of the structure (measured with a power-meter LaserStar (Ophir) coupled with a sensor 3A-P-SH-V1). Responsivity shows an increase from approximatively 2 AW^−1^ to about 7 AW^−1^ depending on the substrate temperature when fabricating the photodetector test structures.

The calculated quantum efficiencies, *QE*, plotted in Figure 7a with dotted lines, have values from 430% for structures prepared at 300 °C to 900% for 500 °C. As a reference, an analogous structure Al/n-Si/SiO_2_/ITO fabricated without Ge-nps in the oxide thin layer shows a very weak absorption, with signals obtained only in the wavelength range specific to Si. This can be considered as a contribution from the substrate and SiO_2_ thin film, this phenomenon been known as negative photoconductivity [41]. Structures with Ge-nps embedded into the SiO_2_ layer exhibit a quite different spectral behavior. As a consequence, the Ge-nps confined in the SiO_2_ matrix play a more important role in the measured broad photoresponsivity spectrum towards low energy (an effect which is amplified as the size of Ge-nps increase) [42–44] compared to the traps formed at the interface between Ge-nps and the surrounding SiO_2_ [45]. Such increased responsivity (leading to *QE* higher than 100%) were also observed and reported for other types of Si- or Ge-based structures [15]. The increased current density and the high spectral photoresponsivity can be the results of carrier multiplication in Ge-nps as suggested also by the analysis conducted by S. Saeed and co-workers [46]. The carrier multiplication can be a possible mechanism associated with excess electron injection induced by the holes trapped in Ge-nps to explain the observed responsivity increase [39]. In Figure 7b, the detectivity (D*) is presented in order to prove the ability of the photodetector structure to detect weak optical signals. This was calculated using the following equation:

[2]
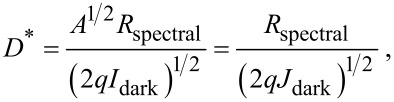


where *A* is the detector area, *q* is the elementary charge, and *I*_dark_ and *J*_dark_ represent the dark current and dark current density, respectively. The detectivity (D*) value is estimated in the range of 10^15^ Jones at about 1040 nm, which is comparable with that of ZnO nanoparticles-based photodetectors (D* ≈ 3.4 × 10^15^ Jones at 360 nm) [47]. The broad wavelength range (from about 450 to 1300 nm) together with the simple fabrication and low-cost process recommend the fabricated photodetector test structure as very promising for future Ge-nps based devices.

**Figure 8:**
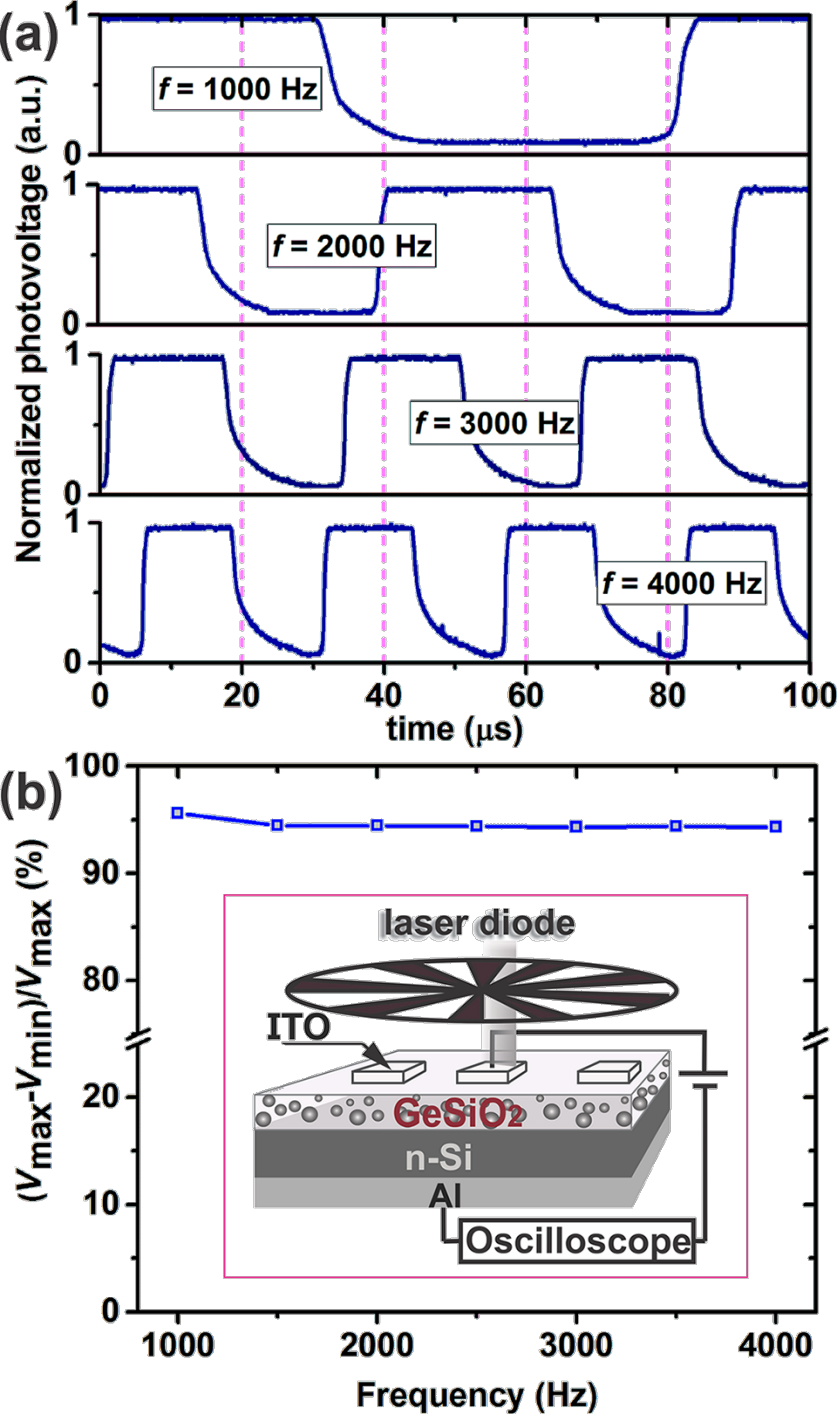
Photovoltaic response speed of the photodetector structure (fabricated at 400 °C) under pulsed (*P*_in_ = 7.6 mW) NIR light (808.5 nm laser): (a) with frequency of 1000, 2000, 3000 and 4000 Hz; (b) the relative balance (*V*_max_ − *V*_min_)/*V*_max_ versus switching frequency. The inset represents the schematic setup used to investigate the time response of the photodetector.

The response speed is one of the most important parameters of a photodetector. As the photocurrents measured at −1 V on samples prepared at 400 and 500 °C have very similar values we present in the following the response speed data obtained on the samples deposited at 400 °C. Figure 8a presents the response speed characteristics of the photodetector test structure fabricated at 400 °C, exposed to pulsed NIR light (808.5 nm) with different frequencies from 1000 to 4000 Hz.

Light modulation was realized using a mechanical chopper and the estimated photovoltage response speed is monitored by an oscilloscope. At zero external bias, an optical incident power of 7.6 mW and a frequency of 4 kHz the estimated rise time (*t*_r_) and fall time (*t*_f_) values are about 0.5 µs and 5.5 µs, respectively. Rise time and fall time were estimated from characteristics taking into consideration only the interval between 10% and 90% of the signal peak value.

For all switching frequency values, the response of the tested photodetector structures is fast and shows a good repeatability even at 4 kHz. Another result that comes to support the improved photodetector properties of the tested structures is the behavior of relative balance versus frequency as plotted in Figure 8b. The relative balance of the tested photodetector structure decreases only by about 1.5% over the whole investigated frequency range (1–4 kHz), which is a good result compared with other new materials and test structures reported in literature (18% in the case of graphene monolayer/germanium heterojunctions or about 13% for MoS_2_/Si heterojunctions) [48–49]. This is an indication of the high quality of the materials deposited by RF-sputtering for this experiment. The results of response time measurements show that photodetector structure as realized here is much quicker than Ge–graphene based photodetectors (*t*_r_ ≈ 23 µs and *t*_f_ ≈ 108 µs) [48], MoS_2_-based photodetectors (*t*_r_ ≈ 3 µs and *t*_f_ ≈ 42 µs) [49] or Ge–graphene–ZnO heterostructure infrared photodetectors (*t*_r_ ≈ 40 µs and *t*_f_ ≈ 90 µs) [50]. More importantly, these are the best results reported so far for photodetectors based on Ge-nps. We attribute this relatively fast response to the extremely high carrier mobility resulting from the high-quality material with high crystallinity, a low density of trap centers, and a quick separation of huge amounts of photogenerated carriers by the built-in electric field formed at the SiO_2_/Ge interface.

## Conclusion

In summary, deposition parameters have been optimized to nanostructure Ge:SiO_2_ thin films during deposition at temperatures lower than those necessary for nanostructuring Ge-nps by thermal annealing after deposition. To optimize the substrate temperature, Al/n-Si/Ge:SiO_2_/ITO photodetector test structures have been fabricated by magnetron sputtering at low temperatures of 300, 400 and 500 °C, and electrical and opto-electrical parameters were investigated and compared. To emphasize the contribution of Ge-nps, an analogous structure Al/n-Si/SiO_2_/ITO was fabricated and the parameters were compared. It was found that the best results were obtained when at a substrate temperature of 500 °C during deposition. The Ge:SiO_2_ layer has a crystalline structure with Ge-nps of about 5 nm randomly distributed within the SiO_2_ thin film. The Al/n-Si/Ge:SiO_2_/ITO photodetector structure fabricated thereof shows very good electrical and opto-electrical parameters at −1 V: a rectification ratio of 10^2^, a photoresponse gain factor of about 10^3^, a responsivity of about 7 AW^−1^, a quantum efficiency of 900%, *t*_r_ and *t*_f_ of 0.5 µs and 5.5 µs, respectively, and a decrease of relative balance of only 1.5%. All these measured properties of the test photodetector structure demonstrate the feasibility of this method for fabricating, in a single step, high-quality Ge-nps embedded in oxide thin films, suitable for applications in optoelectronic devices.

## Experimental

Si:Ge:O thin films have been deposited by magnetron sputtering, co-depositing SiO_2_ and Ge from separate 3 inch targets in a Surrey Nanosystems, Gamma 1000, sputtering equipment. Clean 1 cm^2^ substrates of n-type Si(100) with a resistivity of 10–20 Ω·cm were used and they were firstly degassed for 5 min at 200 °C in vacuum. To obtain the desired Ge/SiO_2_ (50:50) concentration ratio on the Si substrate at a base pressure of 1 × 10^−7^ Torr, the critical deposition parameters were finely tuned around the optimized values of 4 mTorr Ar gas pressure, 30 W DC power for Ge and 262 W RF for SiO_2_, respectively. During deposition the substrate holder was heated to different temperatures of 300–500 °C and rotated for a more uniform thickness. Under these conditions, the resulted film thickness is about 250 nm after 30 min for deposition. For electrical, photoelectrical and speed time response measurements, an aluminium (Al) continuous back electrode and a top matrix of indium thin oxide (ITO) electrodes (9 mm^2^ in size) using the shadow masking technique were deposited by magnetron sputtering (Varian ER3119) and e-beam assisted thermal evaporation (Bestec), respectively. Ge:SiO_2_ films were characterized using an X-ray diffractometer (BRUKER-AXS with Cu Kα_1_ radiation of λ = 0.15406 nm) and an advanced analytical atomic resolution electron microscope (HR-TEM, JEM-ARM200F, JEOL) for structural investigations. Electrical measurements were performed using a set-up containing an optical cryostat (Janis, CCS-450), electrometer (Keithley, 6517A) with built-in DC voltage source and a temperature controller (Lake Shore, 331).

The photoresponse characteristics of the heterojunctions were tested using two light sources. A Xe lamp of 450 W optical power combined with a monochromator (Oriel, 1/4 monochromator) for monochromatic light within the wide range from ultraviolet (UV), visible (vis) to near-infrared (NIR) and an 808.5 nm laser diode used as a stronger light source in the NIR range. Reflectance spectra were obtained using a double beam UV–vis–NIR spectrophotometer (250–3000 nm, Perkin Elmer 950) with specular reflectance accessory (B0086703) at a fixed incidence angle (6°). The response speed of the photodetector test structure was measured by combining a mechanical chopper (Stanford, SR540) and a digital oscilloscope (LeCroy, WaveJet 500 MHz) with a reference photodetector (Thorlabs, PDA10CS-EC).
